# Supplementary material for: The spatial layout of antagonistic brain regions is explicable based on geometric principles
Source: Commun Biol. 2025 Jun 7;8:889. doi: 10.1038/s42003-025-08295-2 (PMC12145436; doi:10.1038/s42003-025-08295-2)
Supplement: Supplementary file 1 — Supplementary Information [file 42003_2025_8295_MOESM1_ESM.pdf]

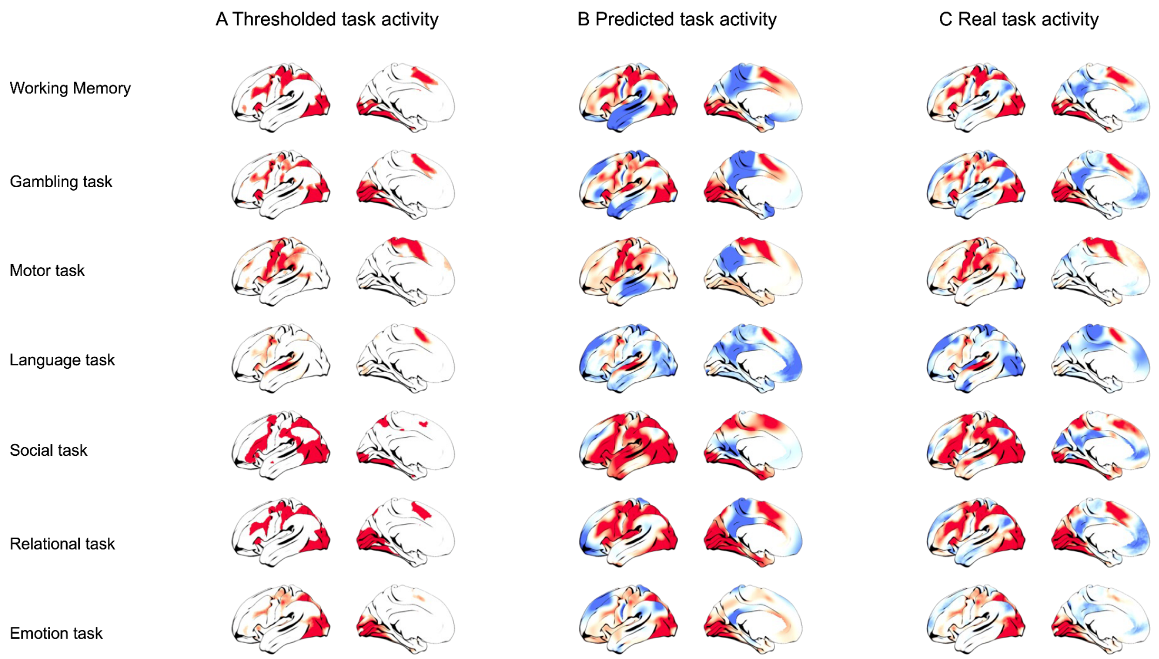

Supplementary figure 1: *Spatial prediction from task-positive activity patterns.* ***A****) Vertices with the highest (> 25%) group-average task activation were used to form a predictor mask.* ***B****) Spatial regression was used to predict task activity at all remaining vertices, and compared to* ***C)*** *the true pattern of activity across all vertices.*

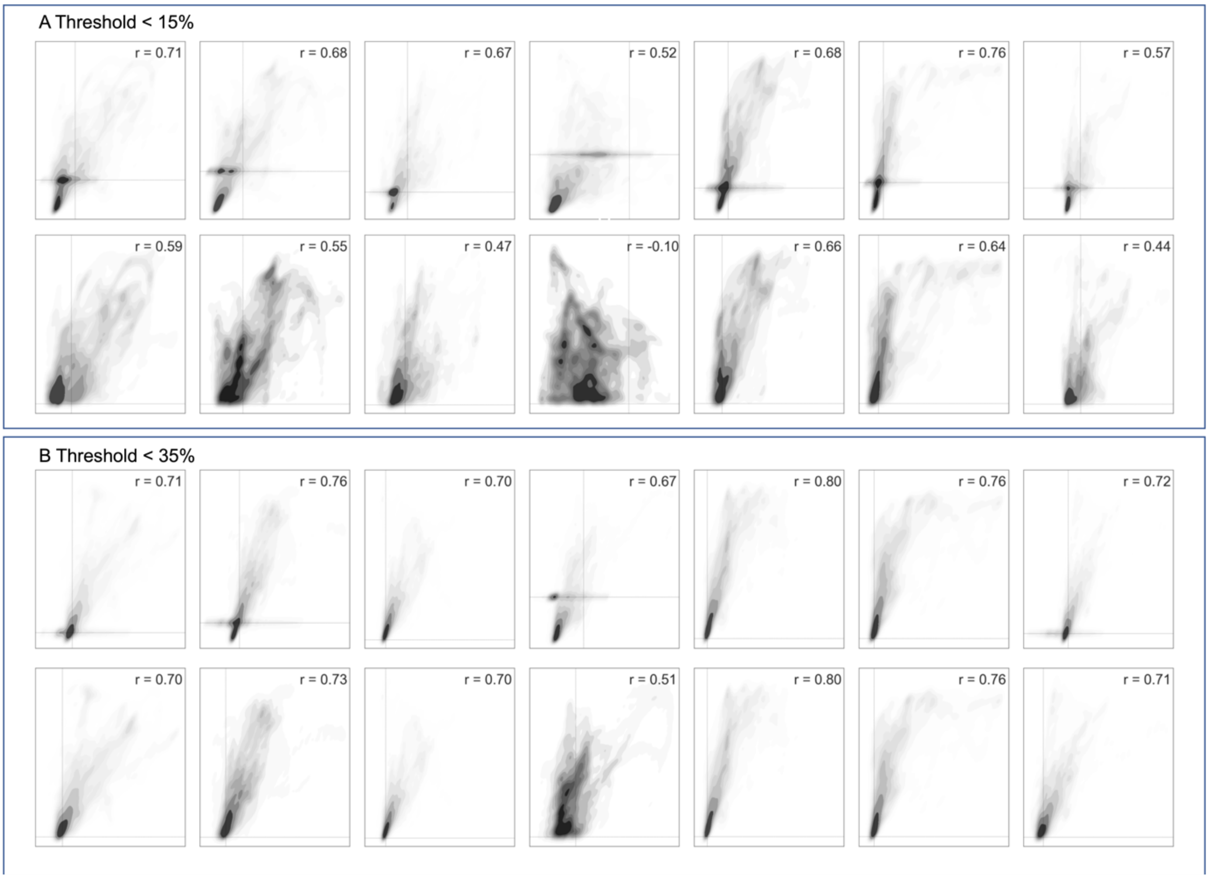

Supplementary figure 2: *Spatial prediction from task-positive activity patterns with different thresholds.* ***A****) Vertices with the lowest (<15%) group-average task activation were used to form a predictor mask: top, all vertices not in predictor mask; bottom, only positive vertices.* ***B****) Vertices with the lowest (<35%) group-average task activation were used to form a predictor mask: top, all vertices not in predictor mask; bottom, only positive vertices.*

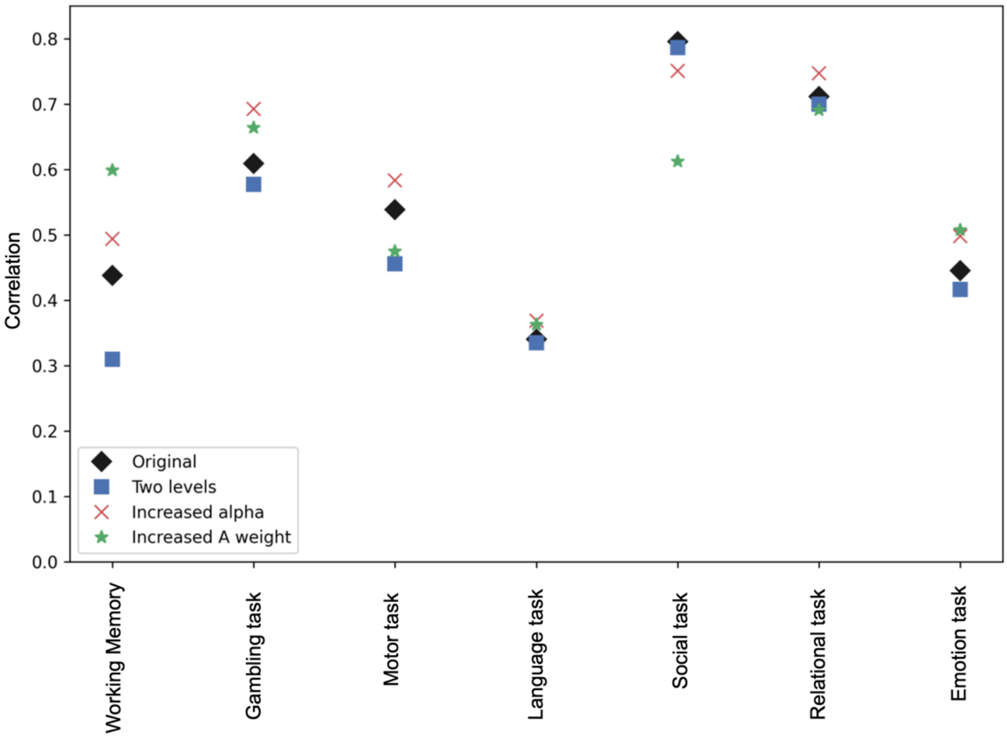

Supplementary figure 3: *Predictive performance on out-of-mask, positive vertices for different LatticeKrig model parameters. Three parameters were varied: (i) the number of levels for the multiresolution lattice (3 in the original or 2); (ii) the alpha parameters controlling the relative variances of the different levels ([1, 0.25, 0.01] for the original or [1, 0.5, 0.1]; (iii) the AWeight (corresponding to the correlation range of the spatial autoregression model) which was either 1.01 in the original or 6.01.*
