# Supplementary material for: The spatial layout of antagonistic brain regions is explicable based on geometric principles
Source: Commun Biol. 2025 Jun 7;8:889. doi: 10.1038/s42003-025-08295-2 (PMC12145436; doi:10.1038/s42003-025-08295-2)
Supplement: Supplementary file 2 — Reporting summary [file 42003_2025_8295_MOESM2_ESM.pdf]

nature portfolio

Corresponding author(s): Robert Leech

Last updated by author(s): Apr 17, 2025

# Reporting Summary

Nature Portfolio wishes to improve the reproducibility of the work that we publish. This form provides structure for consistency and transparency in reporting. For further information on Nature Portfolio policies, see our Editorial Policies and the Editorial Policy Checklist.

## Statistics

For all statistical analyses, confirm that the following items are present in the figure legend, table legend, main text, or Methods section.

| n/a | Confirmed | |
|---|---|---|
| ☐ | ☒ | The exact sample size ($n$) for each experimental group/condition, given as a discrete number and unit of measurement |
| ☐ | ☒ | A statement on whether measurements were taken from distinct samples or whether the same sample was measured repeatedly |
| ☐ | ☒ | The statistical test(s) used AND whether they are one- or two-sided<br>*Only common tests should be described solely by name; describe more complex techniques in the Methods section.* |
| ☒ | ☐ | A description of all covariates tested |
| ☐ | ☒ | A description of any assumptions or corrections, such as tests of normality and adjustment for multiple comparisons |
| ☐ | ☒ | A full description of the statistical parameters including central tendency (e.g. means) or other basic estimates (e.g. regression coefficient) AND variation (e.g. standard deviation) or associated estimates of uncertainty (e.g. confidence intervals) |
| ☐ | ☒ | For null hypothesis testing, the test statistic (e.g. $F$, $t$, $r$) with confidence intervals, effect sizes, degrees of freedom and $P$ value noted<br>*Give P values as exact values whenever suitable.* |
| ☒ | ☐ | For Bayesian analysis, information on the choice of priors and Markov chain Monte Carlo settings |
| ☐ | ☒ | For hierarchical and complex designs, identification of the appropriate level for tests and full reporting of outcomes |
| ☒ | ☐ | Estimates of effect sizes (e.g. Cohen's $d$, Pearson's $r$), indicating how they were calculated |

*Our web collection on statistics for biologists contains articles on many of the points above.*

## Software and code

Policy information about availability of computer code

| | |
|---|---|
| Data collection | No data collection was performed for this study |
| Data analysis | All code for data analysis is provided here: https://github.com/ActiveNeuroImaging/SpatialAnticorrelations |

For manuscripts utilizing custom algorithms or software that are central to the research but not yet described in published literature, software must be made available to editors and reviewers. We strongly encourage code deposition in a community repository (e.g. GitHub). See the Nature Portfolio guidelines for submitting code & software for further information.

## Data

Policy information about availability of data

All manuscripts must include a data availability statement. This statement should provide the following information, where applicable:

- Accession codes, unique identifiers, or web links for publicly available datasets
- A description of any restrictions on data availability
- For clinical datasets or third party data, please ensure that the statement adheres to our policy

No data was collected for this study. Description of open data sources is provided in the manuscript.

## Research involving human participants, their data, or biological material

Policy information about studies with human participants or human data. See also policy information about sex, gender (identity/presentation), and sexual orientation and race, ethnicity and racism.

| | |
|---|---|
| Reporting on sex and gender | The human data are from open source Human Connectome Project. No individual subject data is used. Full details of sex and gender reporting are available at Glasser et al, 2016. |
| Reporting on race, ethnicity, or other socially relevant groupings | The human data are from open source Human Connectome Project. No individual subject data is used. Full details of the demographic characteristic of the group are available at Glasser et al, 2 016. |
| Population characteristics | The human data are from open source Human Connectome Project. No individual subject data is used. Full details of the entire group are available at Glasser et al, 2016. |
| Recruitment | Recruitment information is available at Glasser et al, 2016. |
| Ethics oversight | Details of the institutional review boards and ethical oversight can be found in Glasser et al, 2016 |

Note that full information on the approval of the study protocol must also be provided in the manuscript.

# Field-specific reporting

Please select the one below that is the best fit for your research. If you are not sure, read the appropriate sections before making your selection.

☒ Life sciences ☐ Behavioural & social sciences ☐ Ecological, evolutionary & environmental sciences

For a reference copy of the document with all sections, see nature.com/documents/nr-reporting-summary-flat.pdf

# Life sciences study design

All studies must disclose on these points even when the disclosure is negative.

| | |
|---|---|
| Sample size | Not applicable given the methodology and data used. We were not looking at variability across individuals. The primary dataset is >900 participants and we were looking at group average results. |
| Data exclusions | We excluded no data. |
| Replication | The broad results were replicated in different experimental conditions, and across human and a mouse dataset. |
| Randomization | Not applicable |
| Blinding | Not applicable |

# Reporting for specific materials, systems and methods

We require information from authors about some types of materials, experimental systems and methods used in many studies. Here, indicate whether each material, system or method listed is relevant to your study. If you are not sure if a list item applies to your research, read the appropriate section before selecting a response.

### Materials & experimental systems

| n/a | Involved in the study | |
|---|---|---|
| ☒ | ☐ | Antibodies |
| ☒ | ☐ | Eukaryotic cell lines |
| ☒ | ☐ | Palaeontology and archaeology |
| ☒ | ☐ | Animals and other organisms |
| ☒ | ☐ | Clinical data |
| ☒ | ☐ | Dual use research of concern |
| ☒ | ☐ | Plants |

### Methods

| n/a | Involved in the study | |
|---|---|---|
| ☒ | ☐ | ChIP-seq |
| ☒ | ☐ | Flow cytometry |
| ☐ | ☒ | MRI-based neuroimaging |

## Plants

| | |
|---|---|
| Seed stocks | NA |
| Novel plant genotypes | NA |
| Authentication | NA |

## Magnetic resonance imaging

### Experimental design

| | |
|---|---|
| Design type | Not applicable |
| Design specifications | Not applicable |
| Behavioral performance measures | Not applicable |

### Acquisition

| | |
|---|---|
| Imaging type(s) | Functional |
| Field strength | 3t |
| Sequence & imaging parameters | Details can be found in Glasser et al, 2016 |
| Area of acquisition | Details can be found in Glasser et al, 2016 |

Diffusion MRI ☐ Used ☐ Not used

### Preprocessing

| | |
|---|---|
| Preprocessing software | Details can be found in Glasser et al, 2016 |
| Normalization | Details can be found in Glasser et al, 2016 |
| Normalization template | Details can be found in Glasser et al, 2016 |
| Noise and artifact removal | Details can be found in Glasser et al, 2016 |
| Volume censoring | Details can be found in Glasser et al, 2016 |

### Statistical modeling & inference

| | |
|---|---|
| Model type and settings | Spatial regression (see the manuscript for details). |
| Effect(s) tested | Not applicable |

Specify type of analysis: ☐ Whole brain ☐ ROI-based ☒ Both

Anatomical location(s) *Describe how anatomical locations were determined (e.g. specify whether automated labeling algorithms or probabilistic atlases were used).*

| | |
|---|---|
| Statistic type for inference (See Eklund et al. 2016) | Not relevant (see the manuscript) |
| Correction | Not relevant (see the manuscript) |

## Models & analysis

| n/a | Involved in the study |
| --- | --- |
| ☒ | ☐ Functional and/or effective connectivity |
| ☒ | ☐ Graph analysis |
| ☒ | ☐ Multivariate modeling or predictive analysis |
